# A case report on autoimmune polyglandular syndrome type 2 with pernicious anemia

**DOI:** 10.1002/ccr3.7413

**Published:** 2023-06-08

**Authors:** Niharikha Arram, Romana Riyaz, Sumalatha khatroth, Abhigan Babu Shrestha

**Affiliations:** ^1^ Apollo Institute of Medical Sciences and Research Hyderabad India; ^2^ Shadan Institute of Medical Sciences and Research Hyderabad India; ^3^ Mallareddy Medical College for Women India; ^4^ Department of Internal Medicine M Abdur Rahim Medical College Dinajpur Bangladesh

**Keywords:** Addison's disease, autoimmune polyglandular syndrome type 2, autoimmune thyroiditis, pernicious anemia

## Abstract

Autoimmune polyglandular syndrome type 2 (APS II) is a rare autoimmune disease that affects many endocrine glands. We present a case of a 32‐year‐old man with Addison's disease, autoimmune thyroiditis, and pernicious anemia. Multi‐line and timely management are crucial for each association.

## 1 INTRODUCTION

Autoimmune polyglandular syndromes (APSs) are a rare group of polyendocrine conditions that includes multiple glandular deficiencies associated with other autoimmune diseases,^1^ such as hypergonadotropic hypogonadism, vitiligo, chronic atrophic gastritis, pernicious anemia, chronic autoimmune hepatitis, and celiac disease. And, autoimmune polyendocrine syndrome type 2 (APS II) is defined by the presence of Addison's disease (AD) associated with autoimmune thyroid disease and/or diabetes mellitus (DM) type 1.^2^ This syndrome occurs in approximately 1.4–2 cases per 100,000 inhabitants.^3^ It occurs more commonly in women than men and is generally detected during adolescence.

Here, we present a case of 38 years old man presenting APS II with the complete triad. This case report has been drafted in line with the CARE guideline.^4^

## 2 CASE REPORT

**FIGURE 1:**
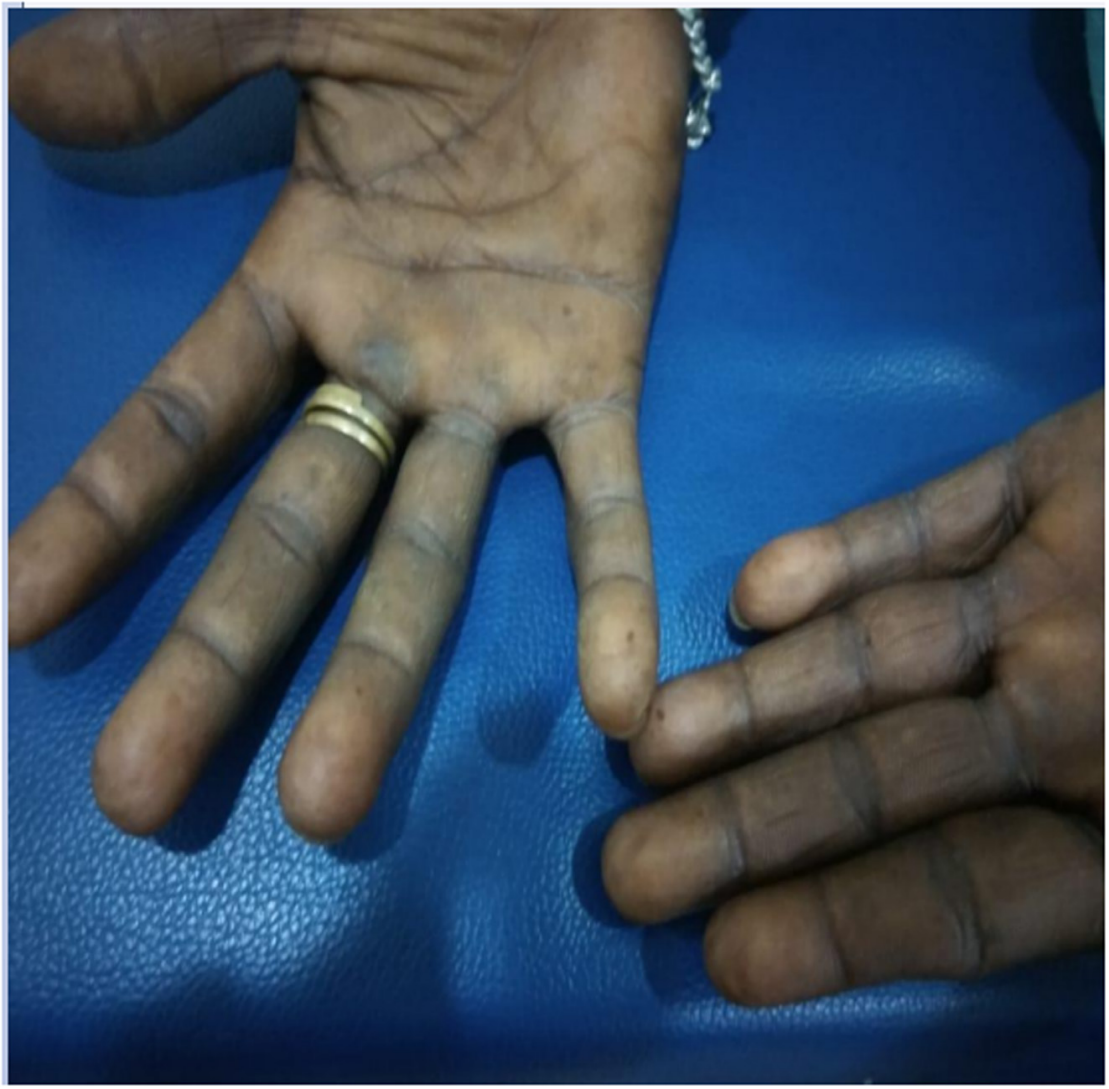
Pallor over palms and hyperpigmented thumbs.

**FIGURE 2:**
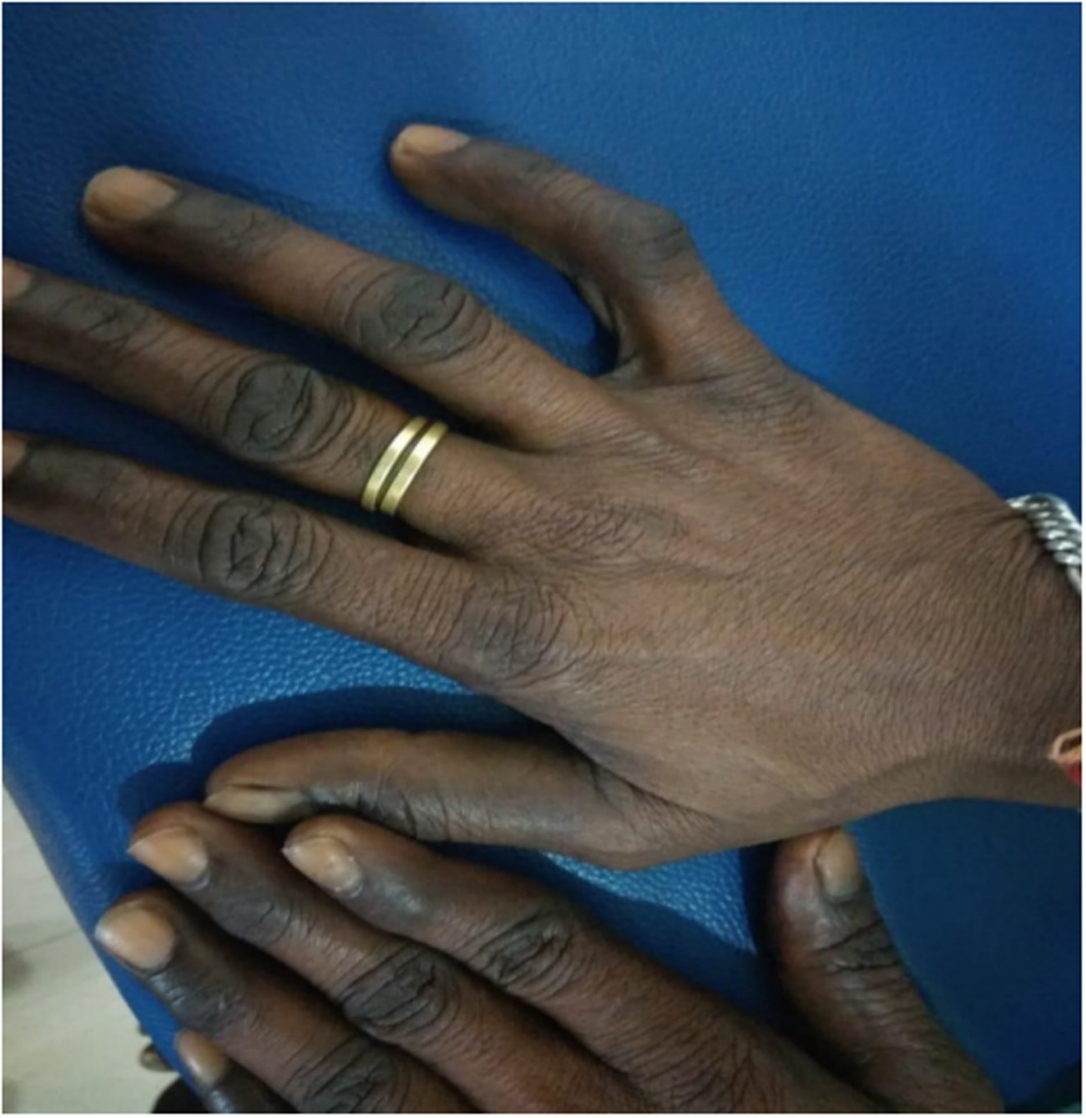
Hyperpigmented knuckles.

**FIGURE 3:**
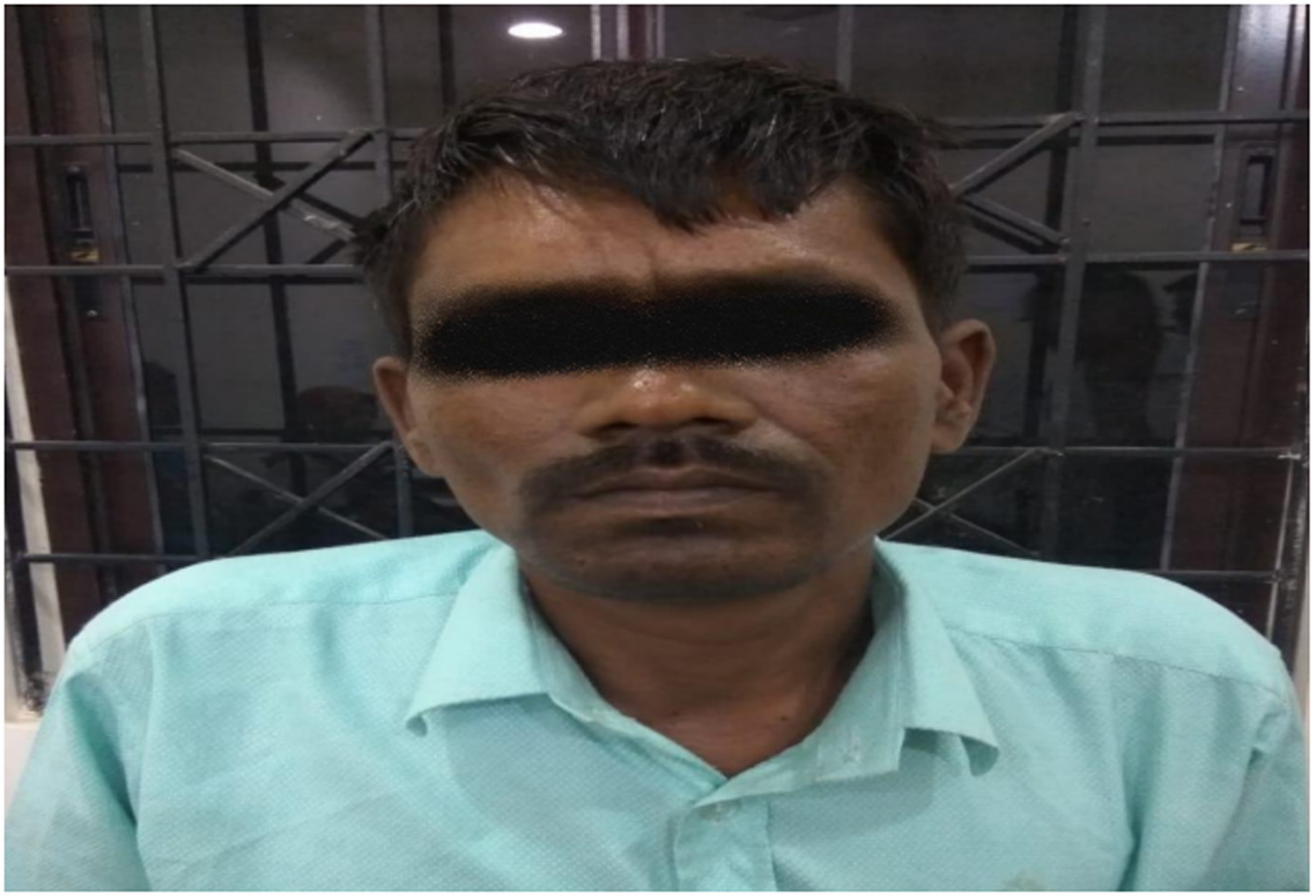
Hyperpigmented lips.

We report a case of a 38‐year‐old man presenting with symptoms of fever for 5 days, associated with generalized weakness, fatigue, and anorexia. Further history revealed that he has had an increased craving for salt. On general examination, pallor, mild icterus, and pigmentation of palms, knuckles, and oral mucosa were noted. Figures 1, 2, 3. None of his family members had similar conditions.

On admission, his blood pressure was 90/60 mm of Hg, pulse rate was 76 beats per minute, and Respiratory rate was 13 times per minute. Blood tests showed low hemoglobin, platelets, and a lower limit of the normal level of White blood cell count (4500/MCL). His serum sodium was decreased, serum potassium was increased, and serum chloride was normal. Urine routine microscopy and examination revealed no abnormalities. In addition to these tests, thyroid function tests revealed increased TSH, normal T3 and T4, and anti‐thyroid peroxidase antibodies were high. Furthermore, Cortisol levels were decreased, and adrenocorticotropic hormone (ACTH) was increased. His CT abdomen was normal. Other tests like Vitamin B12 were decreased (130 pg|mL), Anti‐intrinsic factor antibody was increased (33.6 units) Table 1.

**TABLE 1:** Laboratory findings.

Parameters	Results	Units	Reference values
Random blood sugar	1104	mg/dL	8 80–140
J Hemoglobin	7.2	g/dL	13.8–17.2
Red blood cell	20	million/cumm	3.93–5.69 million cells per cubic millimeter
White blood cell	4500	mcL	4500–11,000
Platelet count	947,000	mcL	150,000‐450,000
Sodium	1125	mmmol/L	1133–146
Potassium	5.8	mmol/L	3.6–5.2
Chloride	102	mmg/dl	0.0.6–1.4
TSH	15.8	mIU/L	0.5–5.0
T3	1.07	nmol/L 0.	0.9–2.8
T4 5.	5.5	μg/dL	5–12
Anti‐TPO antibodies	>1300 i	IU/mL	II <9
U Cortisol	3. 2.27	mmmol/L	2.2.5–7.8
ACTH	74	pg/mL	10–60
Blood urea	15	mg/dL 6	6–24
Creatinine	0.6	mg/dL 0	0.7–1.3
Vitamin B12	V130	pg/mL	160–950
Intrinsic factor antibody	33.6	Aμ/mL	1.21–1.52

All the above features and laboratory tests confirm that the final diagnosis of Addison's disease with Hashimoto's thyroiditis and pernicious anemia, is suggestive of autoimmune polyglandular syndrome type 2. Prompt treatment by Fluids (i.e., regular saline with dextrose) was administered intravenously at twice the maintenance dose. Hydrocortisone 100 mg was administered intravenously. Intramuscularly Vitamin B12 and oral folic acid supplementation were given. The patient was scheduled for a follow‐up visit after a month for further management.

## 3 DISCUSSION

APS is a rare complex disorder of various autoimmune disorders with unknown etiology that are characterized by the co‐occurrence of at least two endocrine deficiencies. Four major categories of APS are known based on clinical features and mode of inheritance APS type 1, APS type 2, APS type 3, and APS type 4. APS type 2 is more common than APS type 11.^2^ However, the association of APS 2 with pernicious anemia is rare, and to the best of our knowledge, only a few cases have been reported previously in the literature.^5^, ^6^

The etiology probably lies in genetic or environmental factors. APS type 2 has a genetic predisposition with alleles of HLA genes, more specifically HLA‐DR3 and HLA‐DR4 contributes to the disease risk and is most commonly inherited in First‐degree relatives.^7^ Subsequently, the presence of one autoimmune condition is rare, progression of one should raise suspicion for both endocrine autoimmune disorders like Addison's disease (50%–70%), Graves disease or autoimmune thyroiditis (15%–69%), type 1 diabetes (41%–50%) and non‐endocrine autoimmune disorders like primary hypogonadism, alopecia, vitiligo and celiac disease.^8^

Primary adrenal insufficiency or Addison's disease results from damage to adrenal glands and disease intrinsic to the adrenal cortex. It is characterized by inadequate production or action of glucocorticoids.^9^ Clinical manifestations include fatigue, weakness, fever, anorexia, nausea, vomiting, hyponatremia, hyperkalemia, metabolic acidosis, and less common symptoms like salt craving, secretory diarrhea, constipation, syncope, and hypoglycemia.^10^ Renal salt excretion, low volume, and excess of antidiuretic hormone (ADH) caused by aldosterone insufficiency are attributable to dilutional hyponatremia. Loss of aldosterone, which normally promotes the urinary excretion of dietary potassium results in hyperkalemia. Adrenal crises result in life‐threatening complications of adrenal insufficiency by a lack of catecholamine action leading to acute hypotension and metabolic alkalosis. Hence, a timely diagnosis with clinical features and investigations is critically important to save patients driving to the adrenal crisis with regular follow‐up. In our patient, these classic abnormalities were observed. Similar history and examination pointed toward Addison's disease, with laboratory tests confirming our diagnosis and suggestive of other autoimmune diseases. Also, he concurrently had Hashimoto's thyroiditis and pernicious anemia.

Autoimmune destruction of insulin‐producing beta cells results in Type 1 diabetes mellitus and is characterized by the presence of insulitis and beta‐cell autoantibodies and one‐third of patients develop an APS. However, our patient did not had any clinical signs and symptoms of diabetes mellitus. It is known that almost 15%–30% of Type1 DM subjects are associated with autoimmune thyroid disease (Hashimoto's or Graves' disease), and 5%–10% are diagnosed with autoimmune gastritis and/or pernicious anemia (AIG /PA), These diseases are characterized by the presence of autoantibodies against thyroid peroxidase (for Hashimoto's thyroiditis), TSH receptor (for Graves' disease), parietal cell or intrinsic factor (for AIG /PA), and 21‐hydroxylase (for AD).^11^

Understanding the polymorphism of syndromes and their disease manifestations leads to early diagnosis and treatment in patients. The purpose of this report is to describe a patient with life‐threatening adrenal cortical insufficiency and autoimmune thyroiditis as autoimmune polyglandular syndrome type 2 subsequently developed pernicious anemia. When acute adrenal insufficiency is diagnosed, the management approach should not be delayed pending laboratory investigation results. In patients with Adrenal crisis, the main goal is to reverse hypotension and electrolyte abnormalities and treat shock immediately. The main line of therapy includes fluids at twice the maintenance dose and intravenous corticosteroids. Some doctors prefer dexamethasone (2–4 mg depending on age) as the effects last 12–14 h and the substitutes do not affect steroid measurements in subsequent ACTH tests. The dose can generally be reduced over 3 days to a maintenance dose of 15–20 mg of hydrocortisone orally. To reduce the adverse effects of weight gain and osteoporosis, the purpose of the lowest dose is to relieve the symptoms. In patients with primary adrenal insufficiency, mineralocorticoids should be replaced with fludrocortisone 0.1 mg.

Our patient developed pernicious anemia due to autoimmune etiology, which led to severe inflammatory reaction and resulted in clinical manifestations of anemia, pigmentation of knuckles, fatigue, and signs of neuropathy. Management by Intramuscular vitamin B12 and oral folic acid supplementation has proven to be effective and his follow‐up after a month revealed lab investigations within normal limits.

Hence, this case report is very important because it delineates all the information needed for the early diagnosis and the corresponding management that physicians need to know for the treatment of the patients to avoid the morbidity of the syndrome.

## 4 CONCLUSION

Adrenal insufficiency is an emergency condition that needs prompt treatment. While the treatment, a physician must always bear the differential of other diseases such as APS type II. Despite being a rare entity, a timely diagnosis, and proper management with long‐term follow‐up are warranted.

## AUTHOR CONTRIBUTIONS

**Niharikha Arram:** Writing – original draft; writing – review and editing. **Romana Riyaz:** Writing – original draft; writing – review and editing. **Sumalatha Khatroth:** Writing – original draft; writing – review and editing. **Abhigan Babu Shrestha:** Writing – original draft; writing – review and editing.

## FUNDING INFORMATION

This research did not receive any specific grant from funding agencies in the public, commercial, or not‐for‐profit sectors.

## CONFLICT OF INTEREST STATEMENT

All authors declare no conflict of interest.

## REGISTRATION OF RESEARCH STUDIES

Not applicable.

## CONSENT

Written informed consent was obtained from the parent's patient for publication of this case report. A copy of the written consent is available for review by the Editor‐in‐Chief of this journal.

## Data Availability

Data sharing not applicable‐no new data generated.
